# Candidemia among COVID-19 patients in Turkey admitted to ICUs: A retrospective multicenter study

**DOI:** 10.1093/ofid/ofac078

**Published:** 2022-02-13

**Authors:** Amir Arastehfar, Nevzat Ünal, Tuğrul Hoşbul, Muhammed Alper Özarslan, Ayşe Sultan Karakoyun, Furkan Polat, Diego Fuentes, Ramazan Gümral, Tuba Turunç, Farnaz Daneshnia, David S Perlin, Cornelia Lass-Flörl, Toni Gabaldón, Macit Ilkit, M Hong Nguyen

**Affiliations:** 1 Center for Discovery and Innovation, Hackensack Meridian Health, Nutley, NJ 07110, USA; 2 University of Health Sciences, Adana City Training and Research Hospital, Laboratory of Medical Microbiology, Adana, 01370, Turkey; 3 Department of Microbiology, Gulhane Training and Research Hospital, University of Health Sciences, Ankara, 06010, Turkey; 4 Department of Microbiology, Faculty of Medicine, Ege University, Izmir, 35100, Turkey; 5 Division of Mycology, Faculty of Medicine, Çukurova University, Adana, 01330, Turkey; 6 Life Sciences Programme, Supercomputing Center (BSC-CNS), Jordi Girona, 08034 Barcelona, Spain; 7 Institute for Research in Biomedicine (IRB Barcelona), The Barcelona Institute of Science and Technology, Baldiri Reixac, 10, 08028 Barcelona, Spain; 8 University of Health Sciences, Adana Faculty of Medicine, Adana City Training and Research Hospital, Department of Infectious Diseases and Clinical Microbiology, Adana, 01370, Turkey; 9 Division of Hygiene and Medical Microbiology, Medical University of Innsbruck, 6020 Innsbruck, Austria; 10 Catalan Institution for Research and Advanced Studies (ICREA), Barcelona, Spain; 11 Centro de Investigación Biomédica en Red de Enfermedades Infecciosas (CIBERINFEC), Barcelona, Spain; 12 Department of Medicine, University of Pittsburgh, Pittsburgh, PA 15261, USA

**Keywords:** COVID-19, candidemia, infection control, bloodstream infection, antibiotic utilization, Candida parapsilosis, fluconazole resistance, Y132F

## Abstract

**Objectives:**

We evaluated the epidemiology of candidemia among COVID-19 patients admitted to intensive care units (ICUs).

**Methods:**

We conducted a retrospective multicenter study in Turkey between April- December 2020.

**Results:**

Twenty-eight of 148 enrolled patients developed candidemia, yielding an incidence of 19% and incidence rate of 14/1,000 patient-days. The probability of acquiring candidemia at 10, 20 and 30 days of ICU admission was 6%, 26% and 50%, respectively. Over 80% of patients received antibiotics, corticosteroid and mechanical ventilation. Receipt of a carbapenem (odds ratio and 95% confidence interval (OR, 95% CI) of 6.0 (1.6–22.3), P=0.008), central venous catheter (4.3 (1.3–14.2), P=0.02) and bacteremia preceding candidemia (6.6 (2.1–20.1), P=0.001) were independent risk factors for candidemia. Mortality rate did not differ between patients with and without candidemia. Age (1.05 (1.01–1.09), P=0.02) and mechanical ventilation (61 (15.8–234.9), P<0.0001) were independent risk factors for death. Candida albicans was the most prevalent species overall. In Izmir, C. parapsilosis accounted for 50% (2/4) of candidemia. Both C. parapsilosis isolates were fluconazole non-susceptible, harbored Erg11-Y132F mutation, and were clonal based on whole-genome sequencing. The two infected patients resided in ICUs with ongoing outbreaks due to fluconazole-resistant C. parapsilosis.

**Conclusions:**

Physicians should be aware of the elevated risk for candidemia among COVID-19 patients requiring ICU care. Prolonged ICU exposure and ICU practices rendered to COVID-19 patients are important contributing factors to candidemia. Emphasis should be placed on heightened infection control in the ICU, and developing antibiotic stewardship strategies to reduce irrational antimicrobial therapy.

## INTRODUCTION

Invasive fungal infections, especially those due to *Candida* spp., are associated with huge economical burdens and high mortality rate.[1] Furthermore, emergence of drug-resistant *Candida* species, such as *Candida auris*, *Candida parapsilosis* and *Candida glabrata*, imposes a growing threat due to limited options of antifungal therapy.[2] The classic risk factors associated with candidemia include leukopenia, chronic renal failure, abdominal surgery, intensive care unit (ICU) stay, central venous catheters (CVCs), mechanical ventilation, and long-term use of corticosteroids.[3] Most recently, COVID-19 pandemic has predisposed millions of patients to secondary infections, including fungal infections.[4-7] Indeed, an increased incidence of candidemia associated with COVID-19 and its associated high mortality rate has been reported.[8-10] Recent outbreaks due to multidrug resistant *C. auris* have also been noted in some centers.[11,12] How COVID-19 patients are at risk for *Candida* infections is not fully understood. The reasons may be multifactorial, including immune dysregulation and organ damage resulting from SARS-CoV-2, acquired immunodeficiency state stemming from immunomodulatory agents administered to treat severe cases of COVID-19, and breach in standard healthcare practices of infection prevention and antibiotic stewardship.[13]

To provide a further insight into candidemia associated with COVID-19, we conducted a multicenter study in Turkey to assess the incidence rate and cumulative risk of ICU-acquired candidemia, identify factors predisposing to candidemia, and evaluate the risk factors for mortality.

Acce

## MATERIALS and METHODS

### Patients

This retrospective study was conducted at three hospitals in Turkey (Adana City Hospital, Adana, Ege University Medical Faculty Hospital, Izmir, and Gülhane Training and Research Hospital, Ankara). From April through December 2020, all patients with COVID-19 admitted to the participating ICUs were included in the study.

### Definitions.

Candidemia was defined as positive blood culture for *Candida* species. *Candida* isolates were speciated using matrix assisted laser desorption ionization-time of flight mass spectrometry at individual centers, however antifungal susceptibility was not performed as per standard of care. For this study, susceptibility testing was performed in accordance with CLSI M60-A3,[14] and non-susceptibility to azole agents was defined according to CLSI breakpoints.[4,7,14,15] Immunosuppressed was defined as presence of an active malignancy or immunocompromised state (primary or secondary due to Human Immunodeficiency virus, hematopoetic stem cell or solid organ transplant, receipt of corticosteroid or other immunosuppressed agents, including biologics). Since steroid was administered to almost all patients in this study, we only included patients receiving steroid (> 20 mg prednisone-equivalent daily for > 10 days) prior to COVID-19 diagnosis in the definition.

### Data collection

We reviewed patients' electronic medical records and collected demographic characteristics and underlying medical conditions. Established risk factors for candidemia from published studies were also collected, including the presence of CVCs (and their duration), mechanical ventilation, renal replacement therapy, and use of antibacterial agents.[3,16] Data on bacterial blood stream infections were also included.

### *ERG11* sequencing

Fluconazole-resistant *C. parapsilosis* isolates were subjected to *ERG11* Sanger sequencing using primers and PCR conditions reported elsewhere.[17]

### Whole genome sequencing (WGS)

The genomes of 2 *C. parapsilosis* isolates from Izmir Hospital (isolates 35 and 37) underwent Illumina WGS using NovaSeq 6000.[18] Eight previously sequenced *C. parapsilosis* isolates, including two clonal pairs recovered from the same patient were included in the analysis of genomic variants for comparison.[19,20] Single Nucleotide Polymorphisms (SNPs) were identified using Freebayes[21] as implemented within the PerSVade version 0.10 pipeline.[18] The genome CDR-317 was used as reference. A multiple correspondence analysis (MCA) was performed as previously described.[19]

### Statistical analysis

Data analyses were conducted using Stata/SE v16.1 (StataCorp LLC, College Station, TX, USA) and GraphPad Prism, version 8.0 (GraphPad Software). Comparisons between 2 groups (candidemia versus no candidemia, and dead versus alive) were performed by Mann-Whitney *U* test for continuous variables and Fisher's exact test for categorical variables. Variables significant by univariate analysis ($P \leq 0.05$) were entered into a backward elimination in logistic regression model to determine independent risk factors for candidemia or death. Kaplan-Meier curves were used to estimate candidemia-free survival and overall survival, and a log-rank test was used to compare curves between groups. Significance was defined as $P \leq 0.05$ (2-tailed).

**Patient Consent Statement**. The study protocol was first approved by the Ministry of Health of Turkey, and then by the local ethical committees of each center (Adana City Hospital (No. 78/1362), Gülhane Training and Research Hospital (No. 2021/164), and Ege University Hospital (No. 21-6.1T/60)). The study protocol conforms to standards currently applied in Turkey. Due to retrospective nature of the study, it does not include factors necessitating patient consent.

## RESULTS

Over the study period, 148 patients were admitted to the ICU (Adana City Hospital, 99 patients, Ege University Hospital, 24 patients, and Gülhane Hospital, 25 patients). Demographics and underlying diseases are presented in Table 1. Twenty-eight COVID-19 patients developed candidemia, yielding a candidemic rate of 19%. This rate was 13-fold higher than the rate of patients without COVID-19 admitted to the ICU in the same study period (1.5%) (Table 2). The probability of acquiring candidemia among COVID-19 patients within 10 days of ICU admission was 6%, but this rate increased to 26% at 20 days and 50% at 30 days (Figure 1). The median duration from hospital admission for COVID-19 to ICU admission was 20 days (interquartile range (IQR): 12–28 days), and from ICU admission to candidemia was 12 days (IQR: 8–18 days) (Figure 1).

*Candida albicans* was the most common species recovered (43%, 12/28), followed by *C. parapsilosis* (25%, 7/28), *C. tropicalis* (21%, 6/28), and *C. glabrata*, *C. krusei* and *C. lusitaniae* (4%, 1 each) (Supplemental Figure 1 and Supplemental Table 1). Forty-thee percent (3/7) of *C. parapsilosis* isolates was non-susceptible to fluconazole (MIC ≥4µg/mL) (Supplemental Tables 1 and 2). All fluconazole-non-susceptible *C. parapsilosis* isolates harbored Y132F mutation in the 14α-demethylase Erg11p. Of note, both fluconazole nonsusceptible *C. parapsilosis* isolates from Izmir (isolates 35 and 37) were recovered from azole-naïve patients who resided in units with an ongoing clonal outbreak of fluconazole-resistant *C. parapsilosis* that started in 2015.[22] To evaluate the genetic relatedness of these two isolates, we performed Illumina whole genome sequencing. For comparison, we included whole genome sequencing data from eight *C. parapsilosis* strains from two previous studies: five isolates from the same patient (including two pairs of clonal isolates) in one study,[19] and three unrelated strains from clinical and environmental sources in the second study.[20] Our genomic analyses demonstrated that the two isolates in this study were closely related. A multidimensional analysis of all 10 isolates showed that the isolates 35 and 37 from the 2 patients in this study are highly related and clustered as close as the two known pairs of clonal isolates[19] (Figure 2), suggesting nosocomial transmission. In fact, the isolates 35 and 37 differed by 261 single nucleotide polymorphisms (SNPs). This SNP difference is within the range of differences found among clonal pairs of isolates from the same patient in our previous study,[19] i.e.115 SNPs in one pair (bsc.1700 and nsc.1701), and 407 SNPs in the second pair (ncc.1701 and tcc.1702). These SNP differences are far lower than the differences found between them and the closest unrelated isolates (i.e. 1183 SNPs between 35 and bsc.1700). In the entire patient cohort, 26% (39/148) had at least one episode of bacteremia. Seventy-one percent (20/28) of patients with candidemia and only 16% (19/120) of patients without candidemia had bacteremia ($P$=0.0001). Furthermore, 31% percent (12/39) of patients had more than one episode of bacteremia: 6 patients had 2 episodes of bacteremia and 6 had 3 episodes of bacteremia. Altogether, 43% (12/28) of patients with candidemia and none of those without candidemia had more than an episode of bacteremia ($P$=0.0001). Bacteremia were most commonly caused by Gram positive bacteria (60%, 34/57), especially coagulase-negative *Staphylococcus* (46%, 26/57) (Supplemental Figure 2).

Univariate analysis identified immunosuppressed status (*P*=0.049), receipt of a carbapenem (*P*=0.025), presence of CVC (*P*<0.0001), bacteremia preceding candidemia (*P*≤0.0001) as risk factors for candidemia (*P*<0.0001; Table 1). The duration of indwelling CVC was significantly longer among patients with candidemia than those without (18.5 versus 8 days, respectively, *P*=0.0001). Using logistic regression analysis, receipt of a carbapenem and bacteremia (overall or due to coagulase-negative *Staphylococcus* or *Acinetobacter baumannii* complex) were independent risk factors for candidemia (Table 1).

Information of antifungal therapy was available for 25 patients. Two patients were not treated with antifungals (one of whom died on the day of candidemia detection). Among the three patients infected with fluconazole-resistant *Candida* isolates, two were initially treated with fluconazole which was then transitioned to amphotericin B and one to an anidulafungin, while the other one was azole-naïve. Among the 19 patients infected with fluconazole-susceptible isolates, 13 were treated with fluconazole, 2 with an echinocandin, and 4 were initially treated with fluconazole which was transitioned to amphotericin B (2 patients) or to an echinocandin (2 patients). The overall in-hospital mortality rate was 84% (125/148). There was no significant difference in mortality rate between patients with or without candidemia (86%, 24/28 versus 84%, 101/120, *P*=1.0). The median time from candidemia diagnosis to death was 14 days (IQR: 4–23 days). Older age (*P*=0.03) and requirement of mechanical ventilation (*P*<0.0001) were independent risk factors for death among critically ill COVID-19 patients in ICU (Table 3).

**DISCUSSION**

This multicenter study of critically ill COVID-19 patients requiring ICU admission identified several important findings. First, 19% of patients developed candidemia, a rate that is higher than previously reported in the literature (range: 2.5 to 14%).[23] This rate was increased by 13-fold among non-COVID-19 patients admitted to the same hospitals in the same time period. Second, COVID-19 patients with candidemia had prolonged hospital stay, with a median time of 12 days from ICU admission to candidemia diagnosis. Our finding is in line with a recent study by the Center for Disease Control and Prevention which showed that most candidemia was acquired more than one week after COVID-19.[7] The estimated cumulative risk for candidemia among our patients increased with longer ICU stay, from 6% at day 10 to 26% at day 20 and 50% at day 30 (Figure 1). Third, the majority of patients with candidemia received antibiotics (99%) and corticosteroid therapy (84%), and required mechanical ventilation (84%), all well-established risk factors for candidemia. Lastly, we identified bacterial bloodstream infection (especially due to coagulase-negative *Staphylococcus* and *A. baumannii* complex), receipt of a carbapenem and presence of CVC as independent risk factors for candidemia. Altogether, our data support the notion that prolonged ICU exposure and healthcare therapeutic interventions of COVID-19 patients were responsible for a higher rate of candidemia observed in our study.

During the COVID-19 pandemic, in order to limit patient contact, hospitals utilized mobile and out-of-room monitoring and device controls and extended dwell intravenous catheters.[24] Such approaches could impact infection control practices. Furthermore, the presence of airborne and contact isolation and cumbersome personal protective equipment might have rendered CVC placement more technically difficult. Along the same line, the increase in ICU patient census might have interfered with routine infection control practice such as surveillance and maintenance of CVC devices and favored utilization of these devices for regular blood draws. All of these factors likely adversely increase the risk of catheter contamination and catheter-associated infection. Indeed, 26% (39/148) of our patients had at least one episode of bacteremia, which were most commonly caused by Gram positive bacteria, especially coagulase-negative *Staphylococcus*, a finding that echoes published studies.[25,26] In our study, 71% (20/28) of candidemic patients had preceding bacteremia, and bacteremia was an independent risk factor for subsequent candidemia. All the candidemic cases in our study were primary blood stream infection, as there was no apparent infection at another site, and 86% (24/28) of these cases was central line associated bloodstream infections (CLABSI). Increase in *Candida*-associated CLABSIs among hospitalized COVID-19 patients has been previously reported.[27]

Over 80% of our patients received broad spectrum antibiotics, corticosteroid and mechanical ventilation, which are all classical risk factors for candidemia.[3,16] Utilization of corticosteroid has significantly improved outcome in severely ill hospitalized patients with COVID-19,[28] and mechanical ventilation is required for patients with COVID-19 associated respiratory failure, thus their use is unlikely to be modifiable. However, the fact that 99% of patients received antibiotics is very concerning. Literature to date showed that bacterial co-infection with COVID-19 occurred in only ~14% of patients in ICU,[29] which is less prevalent than in patients with influenza.[30] Nevertheless, empiric antibiotic prescription in COVID-19 patients was widespread,[31] and similar to our finding, >90% of hospitalized COVID-19 patients were noted to receive empirical antibiotics.[32] As the manifestations of COVID-19 patients with cytokine release syndrome mimic bacterial sepsis, it could be difficult for physicians to withhold antibiotics in this setting.

The association with prolonged ICU acquisition, preceding bacteremia (especially with coagulase-negative *Staphylococcus*), and *Candida* CLABSI, suggest that candidemia is linked to infection control issues. Indeed, breach in infection prevention practices has been linked to outbreaks of *C. auris* throughout the globe.[11,25,33] Furthermore, the high rate of administration of antibacterial agents especially carbapenem (80%) might impact bacterial flora and promoted *Candida* growth,[34] and along with corticosteroid utilization, lead to the selection of *Candida* superinfection in these critically ill and medically complexed COVID-19 patients.

Overall, *C. albicans* was the most common species recovered, followed by *C. parapsilosis* and *C. tropicalis*. Two patients from Izmir were infected with genetically-related fluconazole non-susceptible *C. parapsilosis* isolates carrying a Y132F mutation in Erg11p. The clonal outbreaks due to this particular strain of *C. parapsilosis* have been reported in numerous countries,[35-38] and present a particular challenge as the strains persist and cause outbreaks despite application of disinfectants.[39] Centers dealing with clonal outbreak due to *C. parapsilosis* like ours should closely monitor the emergence of fluconazole-resistance, given its association with poorer outcomes.[39] Recently, *C. parapsilosis* isolates with Erg11-Y132F mutation has also been linked to echinocandin resistance, which further complicates treatment strategy.[40] Since these strains retain susceptibility to amphotericin B,[40] and the efficacy of this agent was shown in *in vivo* study,[41] we recommend amphotericin B as empiric antifungal therapy until susceptibility data are available among centers experiencing problem with fluconazole-resistant *C. parapsilosis*.

The mortality rates of COVID-19 in the ICU ranged from 50–65%, and higher among patients requiring mechanical ventilation. Age and need for mechanical ventilation were predictors for mortality among our patients. Unlike previous studies,[5,7,42] we did not detect a worse outcome among patients with candidemia compared with those without (mortality of 86% vs 84%, respectively). It is possible that, with an overall mortality rate of 84%, it is difficult to distinguish attributable mortality from death caused by candidemia or underlying diseases. Previous studies have suggested that the mortality attributed to candidemia is not significant in a population of patients with high expected mortality.[43] Moreover, in this setting, candidemia might merely a marker for severity of illness. Along this line, our data showed that the mortality rate for patients with candidemia was ~2-fold higher among patients with COVID-19 than those without (84% versus 50%). This finding suggests that COVID-19 may amplify the risk of death due to candidemia.

It is important to acknowledge that our study is limited by its retrospective design, and results may have been influenced by practices and patient populations at our hospitals. Experiences at other centers may differ. Furthermore, the number of candidemia among COVID-19 patients was small, and we do not have detailed clinical data associated with non-COVID-19 candidemic patients during the pandemic for comparison. Nevertheless, our study implicates patient management factors that might have elevated the risk for candidemia among COVID-19 patients requiring ICU care. Our study underscores that prolonged ICU exposure and ICU practices rendered to COVID-19 patients are important contributing factors to candidemia. Emphasis should be placed on heightened infection control protocols in the ICU, as well as developing hospital antibiotic stewardship strategies to reduce irrational antimicrobial utilization. Given the large heterogeneity among ICU COVID-19 patients, it is difficult to evaluate the impact of candidemia on patient's outcome. Research with larger multi-center studies is needed for matching candidemia and control groups in order to derive more accurate estimates of mortality attributable to candidemia.

**Potential Conflicts of Interest**

M. H. N. investigator-initiated research grants from Astellas, Merck, Scynexis, Pulmocide, and Cidara for projects unrelated to this study and served on advisory boards for Astellas, Merck, the Medicines Company, Scynexis, and Shionogi. She is part of the MSG/CDC-funded multicenter study evaluating the epidemiology of invasive fungal infection among patients with COVID-19. D.S.P. receives research support and/or serves on advisory boards for Amplyx, Cidara, Scynexis, N8 Medical, Merck, Regeneron, and Pfizer. C.L.F. received grants, personal fees, ant other from Gilead Sciences, Astellas Pharma, Merck Sharp and Dohme, Basilea, and Angelini. The funders had no role in the design of the study; in the collection, analyses, or interpretation of data; in the writing of the manuscript, or in the decision to publish the results. The other authors declare no conflict of interest.

**Funding**

MHN was funded by the National Institute of Health R21 AI160098 and R21 AI152018.

**Table 1. Demographics, clinical characteristics, and factors associated with candidemia**

| | All patients (*n*= 148) | Candidemia (*n*= 28) | No candidemia (*n*= 120) | Univariate analysis *P*-values | Logistic regression #1 *P*-values Odds ratio (95% CI) | Logistic regression #2 *P*-values Odds ratio (95% CI) |
|---|---|---|---|---|---|---|
| **Demographics and underlying diseases** | | | | | | |
| Age in years, median (IQR) | 68 (60–76) | 65 (50–73) | 70 (60–78) | 0.13 | | |
| Male sex | 65% (96) | 75% (21) | 62% (75) | 0.77 | | |
| Cerebrovascular accident | 2% (3) | 7% (2) | 0.8% (1) | 0.09 | | |
| Cardiovascular disease | 19% (28) | 18% (5) | 19% (23) | 1.0 | | |
| Chronic lung disease | 14% (33) | 24% (29) | 18% (22) | 0.32 | | |
| Chronic intestinal disease | 6% (9) | 14% (4) | 4% (5) | 0.07 | | |
| Chronic liver disease | 2% (3) | 0% | 3% (3) | 1.0 | | |
| Chronic kidney disease | 19% (28) | 14% (4) | 20% (24) | 0.60 | | |
| Solid organ malignancy | 13% (19) | 21% (6) | 11% (13) | 0.20 | | |
| Hematologic malignancy | 3% (5) | 7% (2) | 3% (3) | 0.24 | | |
| Immuno-compromised status | 18% (26) | 32% (9) | 14% (17) | 0.049 | 0.34 | 0.75 |
| Hypertension | 51% (75) | 39% (11) | 53% (64) | 0.21 | | |
| Diabetes mellitus | 35% (52) | 21% (6) | 38% (46) | 0.12 | | |
| No known underlying disease | 11% (17) | 7% (2) | 12% (15) | 0.53 | | |
| **Events occurred while inpatient** | | | | | | |
| Receipt of any antimicrobial agent | 99% (147) | 100% (28) | 99% (119) | 1.0 | | |

| | | | | | | |
|---|---|---|---|---|---|---|
| Fluoroquinolones | 70% (103) | 75% (21) | 68% (82) | 0.65 | | |
| Carbapenems[1] | 68% (100) | 86% (24) | 63% (76) | 0.025 | 0.008<br>6.0 (1.61–22.3) | 0.02<br>6.2 (1.4–28.1) |
| Anti-pseudomonal agents (β-lactam, monobactam or carbapenem) | 78% (114) | 67% (18) | 80% (96) | 0.20 | | |
| β-lactam agents without anti-pseudomonal coverage | 12% (17) | 4% (1) | 13% (16) | 0.31 | | |
| Glycopeptide/ oxazolidinone/ cyclic lipopeptide class | 49% (72) | 54% (14) | 48% (58) | 0.67 | | |
| Polymyxin class | 22% (32) | 31% (8) | 20% (24) | 0.29 | | |
| Receipt of corticosteroid | 84% (125) | 71% (20) | 88% (105) | 0.045 | | |
| Presence of central venous catheter | 53% (78) | 86% (24) | 45% (54) | <0.0001 | 0.017<br>4.3 (1.3–14.2) | 0.11 |
| Duration of central venous catheter, days (median, IQR) | 11 days (5–18) | 18.5 days (10–25) | 8 days (4–15) | 0.0001 | | |
| Requirement for mechanical intubation | 84% (125) | 89% (25) | 83% (100) | 0.57 | | |
| Shock requiring pressor support | 81% (104) | 75% (21) | 82% (83) | 0.422 | | |
| **Bacteremia** | | | | | | |
| Preceding bacteremia[2] | 26% (39) | 71% (20) | 16% (19) | <0.0001 | 0.001<br>6.6 (2.1–20.1) | |
| Preceding bacteremia due to coagulase-negative *Staphylococcus* | 15% (24) | 50% (14) | 9% (11) | 0.0001 | | <0.0001<br>13.5 (3.3–55.3) |

| | | | | | |
|---|---|---|---|---|---|
| Preceding bacteremia due to *Acinetobacter baumannii* complex | 9% (13) | 39% (11) | 2% (2) | <0.0001 | <0.0001<br>48.1 (5.9–391.0) |

[1] Among patients receiving carbapenem, all received meropenem; 2 patients received both meropenem and ertapenem but at different times.

[2] Please refer to Supplemental Figure 2 for enumeration of specific bacteria responsible for bloodstream infections among patients with and without bacteremia

**Table 2. Characteristics of centers participated in the current study.**

| | Adana City Hospital | Ege University Medical Faculty Hospital, Izmir | Gulhane Training and Research Hospital, Ankara |
|---|---|---|---|
| Number of hospital beds | 1,550 | 1,800 | 1,350 |
| Number of ICU beds | 310 | 322 | 140 |
| Number of candidemia cases before and after Covid-19 periods[§] | 135 and 246 | 131 and 148 | 75 and 90 |
| Rate of candidemia before and after Covid-19 periods[§] | 3.4% and 19.2% | 1.1% and 16.7% | 1.5% and 17.9% |
| Mortality rates of patients with candidemia before and after Covid-19 periods[§] | 59% and 95% | 42% and 50% | 49% and 80% |

[§]The timeline before COVID-19 period was between January 01, 2019–December 31, 2019, and during Covid-19 period was between April 01, 2020–March 31, 2021.

**Table 3. Factors associated with mortality.**

| Factors | Factors present | Factor absent | Univariate analysis *P*-values | Logistic regression *P*-values | Odds ratio (95% CI) |
|---|---|---|---|---|---|
| Male sex | 85% (82/96) | 83% (43/52) | 0.64 | | |
| No known underlying diseases | 75% (12/16) | 86% (113/132) | 0.28 | | |
| Renal disease | 96% (27/28) | 82% (98/120) | 0.08 | | |
| Hypertension | 88% (65/74) | 80% (60/74) | 0.36 | | |
| Diabetes | 77% (41/53) | 88% (84/95) | 0.10 | | |
| Underlying immunosuppression | 96% (25/26) | 82% (100/122) | 0.08 | | |
| Previous bacteremia | 84% (26/31) | 85% (99/117) | 1.0 | | |
| Receipt of steroid | 86% (107/125) | 78% (18/23) | 0.36 | | |
| Candidemia | 86% (24/28) | 84% (101/120) | 1.0 | | |
| *Candida albicans* | 83% (10/12) | 88% (14/16) | 1.0 | | |
| *Candida parapsilosis* | 86% (6/7) | 86% (18/21) | 1.0 | | |
| Requirement of mechanical ventilation | 95% (119/125) | 26% (6/23) | <0.0001 | <0.0001 | 61 (15.8–234.9) |
| | **Died** | **Alive** | | | |
| Median age, years (IQR) | 70 (61–78) | 60 (40–72) | 0.03 | 0.02 | 1.05 (1.01–1.09) |

**Figure 1.** Time from ICU admission to candidemia among patients with COVID-19. The tick marks indicate censored data. The probabilities of candidemia-free survival at 10, 20, and 30 days are presented above the Kaplan-Meier curve.

**Figure 2.** SNP-based multiple correspondence analysis plot showing genetic clusters based on whole genome SNP analysis. Isolates 35 and 37 were recovered from two patients from Izmir hospital with *C. parapsilosis* fungemia. CBS1954, CBS6318, GA1, and CDC317 represent unrelated clinical and environmental isolates taken from [15]. The remaining five isolates correspond to serial isolates from the same patients where three distinct clonal complexes were identified [14]. Strains considered from the same clonal complex are circled.

Figure 1

Number of patients: 148 137 116 90 76 56 47 40

Figure 2

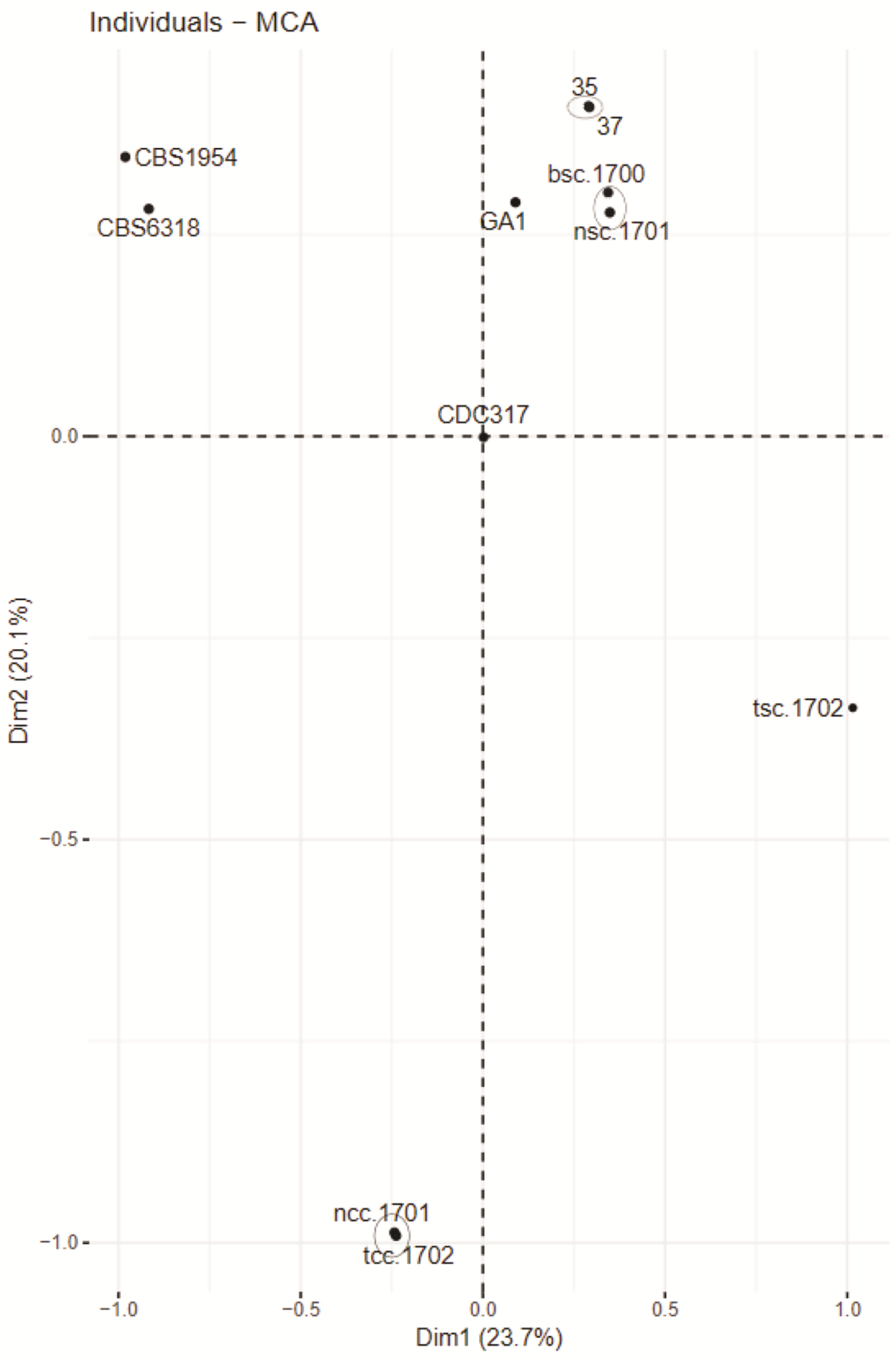

Individuals – MCA
35
37
CBS1954
CBS6318
GA1
bsc.1700
nsc.1701
CDC317
tsc.1702
ncc.1701
tcc.1702
0.0
−0.5
−1.0
−1.0
−0.5
0.0
0.5
1.0
Dim1 (23.7%)
Dim2 (20.1%)

## Supplementary Material

ofac078_suppl_Supplementary_Figures

ofac078_suppl_Supplementary_Tables

