# Supplementary material for: Candidemia Among Coronavirus Disease 2019 Patients in Turkey Admitted to Intensive Care Units: A Retrospective Multicenter Study
Source: Open Forum Infect Dis. 2022 Feb 13;9(4):ofac078. doi: 10.1093/ofid/ofac078 (PMC8903397; doi:10.1093/ofid/ofac078)

**Supplemental Figure 1. *Candida* species recovered from patients with candidemia, stratified by hospitals.**


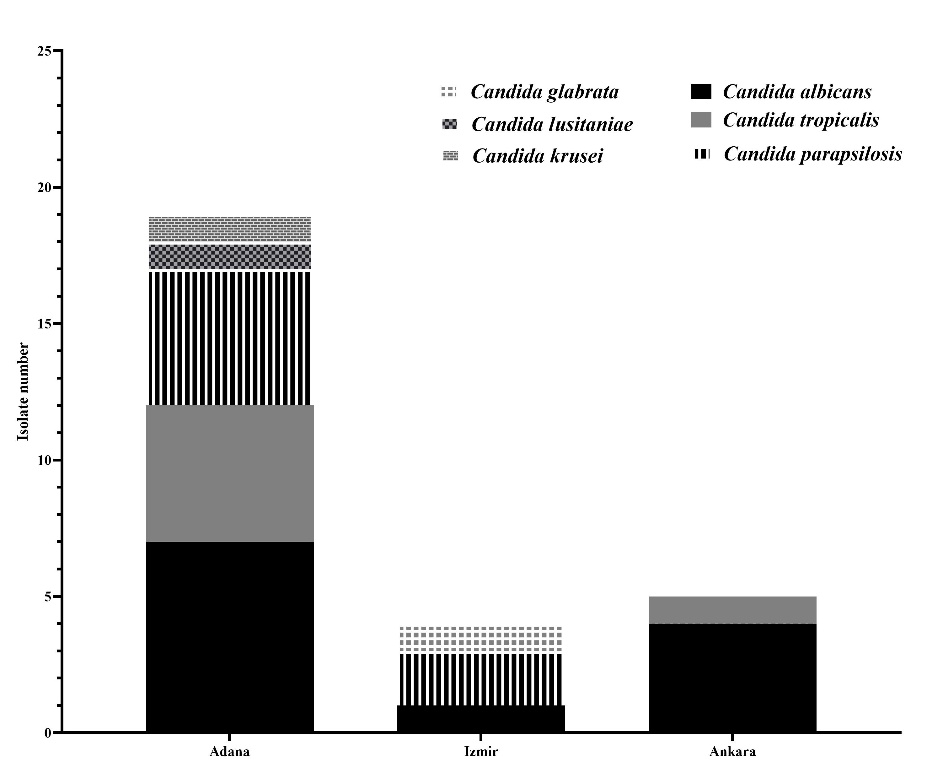


**Supplemental Figure 2. Bacteria species causing blood stream infection, stratified by patients with candidemia (left panel) or without (right panel).** Note that 43% (12/28) of patients with candidemia and none of those without had more than an episode of bacteremia (*P*=0.0001)


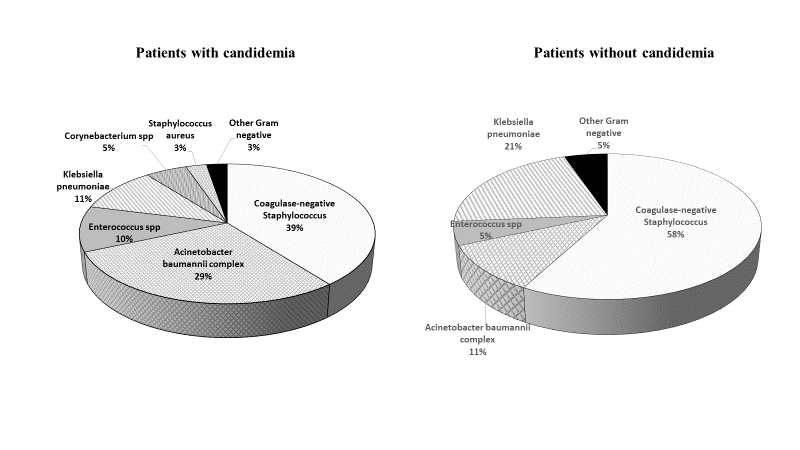

Supplement: ofac078_suppl_Supplementary_Figures [file ofac078_suppl_supplementary_figures.docx]
