# Supplementary material for: Candidemia Among Coronavirus Disease 2019 Patients in Turkey Admitted to Intensive Care Units: A Retrospective Multicenter Study
Source: Open Forum Infect Dis. 2022 Feb 13;9(4):ofac078. doi: 10.1093/ofid/ofac078 (PMC8903397; doi:10.1093/ofid/ofac078)
Supplement: ofac078_suppl_Supplementary_Tables [file ofac078_suppl_supplementary_tables.docx]

**Supplemental Table 1.** **Antifungal susceptibility profile of *Candida* isolates recovered from the blood samples of the COVID-19 patients**

| **Species** |  | **Fluconazole** | **Voriconazole** | **Posaconazole** | **Micafungin** | **Anidulafungin** | **Amphotericin B** |
| --- | --- | --- | --- | --- | --- | --- | --- |
| ***C. albicans* (*n*= 12)** | **S** | 12 | 12 | 12 | 12 | 12 | 12 |
|  | **R** | 0 | 0 | 0 | 0 | 0 | 0 |
|  | **NWT** | 0 | 0 | 1 | 0 | 0 | 0 |
|  | **WT** | 12 | 12 | 11 | 12 | 12 | 12 |
| ***C. parapsilosis* (*n=* 7)** | **S** | 4 | 5 | 7 | 7 | 7 | 7 |
|  | **R** | 2 | 0 | 0 | 0 | 0 | 0 |
|  | **I** | 1 | 2 | 0 | 0 | 0 | 0 |
|  | **NWT** | 3 | 3 | 0 | 0 | 0 | 0 |
|  | **WT** | 4 | 4 | 7 | 7 | 7 | 7 |
| ***C. tropicalis* (*n=* 6)** | **S** | 6 | 6 | 6 | 6 | 6 | 6 |
|  | **R** | 0 | 0 | 0 | 0 | 0 | 0 |
|  | **NWT** | 0 | 0 | 0 | 0 | 0 | 0 |
|  | **WT** | 6 | 6 | 6 | 6 | 6 | 6 |
| ***C. glabrata* (*n=* 1)** | **SDD/S** | 1 | NA | NA | 1 | 1 | 1 |
|  | **R** | 0 | NA | NA | 0 | 0 | NA |
|  | **NWT** | 0 | 0 | 1 | 0 | 0 | 0 |
|  | **WT** | 1 | 1 | 0 | 1 | 1 | 1 |
| ***Pichia kudriavzevii* (*n=* 1)^A^** | **NWT** | 0 | 0 | 0 | 0 | 0 | 0 |
|  | **WT** | 1 | 1 | 1 | 1 | 1 | 1 |
| ***Clavispora lusitaniae* (*n=* 1)^B^** | **NWT** | 0 | 0 | 0 | 0 | 0 | 0 |
|  | **WT** | 1 | 1 | 1 | 1 | 1 | 1 |

A, Equivalent to *Candida krusei*; B. Equivalent to *Candida lusitaniae*; S, Susceptible; R, Resistant; I, Intermediate; SDD, Susceptible dose-dependent; WT, Wild-type; NWT, Non-wild-type.

**Supplemental Table 2.** **Minimum inhibitory concentration of *Candida* isolates recovered from the blood samples of the COVID-19 patients**

| **Species (number of isolates)** | **Antifungal drug** | **Minimum inhibitory concentration (µg/ml)** | | | | | | | | | | | | |
| --- | --- | --- | --- | --- | --- | --- | --- | --- | --- | --- | --- | --- | --- | --- |
|  |  | 0.015 | 0.03 | 0.06 | 0.125 | 0.25 | 0.5 | 1 | 2 | 4 | 8 | 16 | 32 | ≥ 64 |
| ***C. albicans* (*n*= 12)** | **FLZ** |  |  |  | 6 | 5 | 1 |  |  |  |  |  |  |  |
|  | **VRZ** | 11 |  |  |  |  |  |  |  |  |  |  |  |  |
|  | **PSZ** | 11 |  |  | 1 |  |  |  |  |  |  |  |  |  |
|  | **MICA** | 12 |  |  |  |  |  |  |  |  |  |  |  |  |
|  | **ANI** | 12 |  |  |  |  |  |  |  |  |  |  |  |  |
|  | **AMB** |  |  | 5 | 6 | 1 |  |  |  |  |  |  |  |  |
| ***C. parapsilosis* (*n=* 7)** | **FLZ** |  |  |  |  | 1 | 3 |  |  | 1 | 2 |  |  |  |
|  | **VRZ** | 2 | 2 |  | 1 | 1 | 1 |  |  |  |  |  |  |  |
|  | **PSZ** | 5 | 2 |  |  |  |  |  |  |  |  |  |  |  |
|  | **MICA** |  |  |  |  | 1 | 6 |  |  |  |  |  |  |  |
|  | **ANI** |  |  |  |  | 1 |  | 5 | 1 |  |  |  |  |  |
|  | **AMB** |  | 2 | 4 | 1 |  |  |  |  |  |  |  |  |  |
| ***C. tropicalis* (*n=* 6)** | **FLZ** |  |  |  | 5 | 1 |  |  |  |  |  |  |  |  |
|  | **VRZ** | 5 | 1 |  |  |  |  |  |  |  |  |  |  |  |
|  | **PSZ** | 5 | 1 |  |  |  |  |  |  |  |  |  |  |  |
|  | **MICA** | 5 | 1 |  |  |  |  |  |  |  |  |  |  |  |
|  | **ANI** | 6 |  |  |  |  |  |  |  |  |  |  |  |  |
|  | **AMB** |  |  | 1 | 3 | 2 |  |  |  |  |  |  |  |  |
| ***C. glabrata* (*n=* 1)** | **FLZ** |  |  |  |  |  |  |  |  | 1 |  |  |  |  |
|  | **VRZ** |  |  | 1 |  |  |  |  |  |  |  |  |  |  |
|  | **PSZ** |  |  |  |  |  |  | 1 |  |  |  |  |  |  |
|  | **MICA** | 1 |  |  |  |  |  |  |  |  |  |  |  |  |
|  | **ANI** | 1 |  |  |  |  |  |  |  |  |  |  |  |  |
|  | **AMB** |  |  |  |  |  | 1 |  |  |  |  |  |  |  |
| ***Pichia kudriavzevii* (*n=* 1)^A^** | **FLZ** |  |  |  |  |  |  |  |  |  |  | 1 |  |  |
|  | **VRZ** |  |  |  | 1 |  |  |  |  |  |  |  |  |  |
|  | **PSZ** |  |  |  |  | 1 |  |  |  |  |  |  |  |  |
|  | **MICA** |  |  |  | 1 |  |  |  |  |  |  |  |  |  |
|  | **ANI** | 1 |  |  |  |  |  |  |  |  |  |  |  |  |
|  | **AMB** |  |  |  |  |  |  | 1 |  |  |  |  |  |  |
| ***Clavispora lusitaniae* (*n=* 1)^B^** | **FLZ** |  |  |  |  | 1 |  |  |  |  |  |  |  |  |
|  | **VRZ** | 1 |  |  |  |  |  |  |  |  |  |  |  |  |
|  | **PSZ** | 1 |  |  |  |  |  |  |  |  |  |  |  |  |
|  | **MICA** |  |  | 1 |  |  |  |  |  |  |  |  |  |  |
|  | **ANI** | 1 |  |  |  |  |  |  |  |  |  |  |  |  |
|  | **AMB** |  |  |  | 1 |  |  |  |  |  |  |  |  |  |

A, Equal to *Candida krusei*; B, Equal to *Candida lusitaniae*; FLZ, Fluconazole; VRZ, Voriconazole; PSZ, Posaconazole; MICA, Micafungin; ANI, Anidulafungin; AMB, Amphotericin B.
